# Association between sedentary time and sleep quality based on the Pittsburgh Sleep Quality Index among South Korean adults

**DOI:** 10.1186/s12889-021-12388-y

**Published:** 2021-12-15

**Authors:** Sung Hoon Jeong, Bich Na Jang, Seung Hoon Kim, Gyu Ri Kim, Eun-Cheol Park, Sung-In Jang

**Affiliations:** 1 Department of Public Health, Graduate School, Yonsei University, Seoul, Republic of Korea; 2 Institute of Health Services Research, Yonsei University, 50 Yonsei-ro, Seodaemun-gu, Seoul, 03722 Republic of Korea; 3 Department of Preventive Medicine, Yonsei University College of Medicine, 50 Yonsei-ro, Seodaemun-gu, Seoul, 03722 Republic of Korea

**Keywords:** Sedentary time, Insomnia, Sleep disorder, Sleep efficiency, Pittsburgh sleep quality index

## Abstract

**Background:**

Sleep problems increase the risk of premature illness and death. We evaluated the association between sedentary time and sleep quality among South Korean adults.

**Methods:**

The data of adults (aged ≥ 19 years) from the 2018 Korea Community Health Survey were analyzed. Sedentary time, which included hours spent sitting or lying down daily, was categorized into four standardized groups. Poor sleep quality was defined using the global cutoff point (> 5 points) of the Pittsburg Sleep Quality Index. Multiple logistic regression analyses were performed to identify the association between sedentary time (≤ 3.9, 4.0–5.9, 6.0–7.9, and ≥ 8 hours /day) and sleep quality, by sex.

**Results:**

Of the 224,118 participants, 35,784/100,454 men (35.6%) and 58,271/123,664 women (47.1%) had poor sleep quality. Compared with ≤ 3.9 h/day, sedentary times 4.0–5.9, 6.0–7.9, and ≥ 8 h/day were associated with worse sleep quality among men (odds ratio [OR]: 1.12, 95% confidence interval [CI]: 1.08–1.16; OR: 1.19, 95% CI: 1.14–1.25; OR: 1.30, 95% CI: 1.25–1.34, respectively) and women (OR: 1.06, 95% CI: 1.03–1.10; OR: 1.12, 95% CI: 1.08–1.16; OR: 1.22, 95% CI: 1.18–1.26, respectively). In subgroup analyses of sleep quality, subjective sleep quality, latency, disturbance, use of sleeping medication, and daytime dysfunction showed a strong dose-response relationship with increasing sedentary time in both sexes.

**Conclusions:**

Regardless of sex, the longer the sedentary time, the stronger the association with poor sleep quality. Nationwide efforts are required to recommend standards for sedentary time and develop evidenced-based healthy behavior guidelines.

## Background

Insomnia is a common sleep disorder affecting approximately 10–20% of people worldwide. The World Health Organization recently identified poor sleep quality (SQ) as a public health problem that increases the risk of premature disease and death [1, 2]. Poor SQ is characterized by long sleep delays, low sleep efficiency, and sleep disorders [3].

Poor SQ causes health problems such as poor cardiovascular and metabolic function, depression, and other changes in mental health conditions [4, 5]. Deterioration in health makes life unstable, resulting in increased health cost burdens [6]. SQ deterioration, including insomnia, is a commonly reported symptom, especially in primary healthcare settings [7]. Nevertheless, sleep problems often remain undiagnosed and untreated [6], leading to the use of self-prescribed sleeping pills, including melatonin pills, and alcohol use [8]. The frequent use of self-prescribed drugs can increase the resistance to or dependence on substances over time and worsen sleep problem [9]. Provision of cognitive or behavioral therapy by trained professionals to treat sleep problems can be expensive and unaffordable [10, 11]. Therefore, treatments for sleep problems are needed that are low-cost, nonpharmacological, have a wide coverage, and can be easily administered [10]. Increasing physical activity and reducing sedentary time (ST) may be a cost-effective strategy to address this problem.

ST is consistently associated with progression to diabetes, metabolic syndrome, and other chronic diseases, regardless of sufficient physical activity [12]. Various suggestions have been made to reduce ST in the general population, which is a main cause of death [13]. Although the relationship among physical activity, mental health, and SQ is relatively well established [14], the relationship between ST and SQ remains unclear. The relationship of ST with sleep amount is reported only in few studies [15, 16]. A study suggested robust associations of poor SQ with poor functioning, regardless of ST, in the general population [17]. Therefore, it is necessary to study the relationship between SQ and ST, a comprehensive concept covering sleep duration and efficiency.

The Pittsburgh Sleep Quality Index (PSQI) is a widely used tools for measuring SQ [11, 18]. The PSQI, developed in 1989 by Buysse [2] and colleagues, is a self-reporting tool used for evaluating the quality and patterns of sleep over a month [3]. Existing literature on the PSQI provides information on its psychometric properties, internal consistency, test-retest reliability, validity, and factor structure [19, 20].

A few studies have evaluated the association between SQ and ST, focusing on older adults [18] and college students [21]. However, research on the relationship between SQ and ST remains insufficient. Thus, this study investigated the association between ST and SQ in the adult South Korean population using the PSQI.

## Methods

### Data and study population

For this study, we used raw data from the 2018 Korea Community Health Survey (KCHS). The Korea Centers for Disease Control and Prevention conducts this survey every year since 2008. At the time of the survey, trained investigators visited sampled households containing adults aged ≥ 19 years to conduct a 1:1 interview using a laptop equipped with a survey program [22]. The KCHS data are published with open access. Additionally, participants’ information was fully anonymized and deidentified before analysis.

Of the 228,558 participants considered for inclusion in this study, we excluded those who answered, “don’t know,” provided invalid responses to the questions, or did not answer all the questions included in this study (*n* = 4,440). Finally, a total of 224,118 participants (100,454 men and 123,664 women) were selected. Based on a previous study that reported a significant difference in SQ by sex (due to differences in the physiological levels of sex hormones), we performed analyses stratified by sex [23].

### Variables

#### Dependent variable

Poor SQ was defined using the PSQI, a 19-item self-reported questionnaire. The 19 items are divided into the following seven factors: subjective SQ, sleep latency, sleep duration, sleep efficiency, sleep disturbances, use of sleep medication, and daytime dysfunction; each component is scored on a scale ranging from 0 to 3. The SQ score is calculated by adding the scores of all components and ranges from 0 to 21. The PSQI has been widely applied in the general population; the PSQI-Korean version has demonstrated high sensitivity and specificity [20]. However, various studies conducted in Korea provided different cutoff points for SQ, unlike the global cutoff point of a score of 5 or more [20, 24]. In this study, the global cutoff point was applied, with scores of ≤ 5 points and >5 points defined as good and poor SQ, respectively. This cutoff has generally been used in various studies in the Korean population [25, 26], which makes it possible to discriminate transcultural differences.

#### Independent variable

ST was evaluated using the KCHS exercise and physical activity categories: How many hours/minutes (except sleep) did you spend sitting or lying down in a normal day? This included time spent sitting on chairs or benches to watch television on weekdays or weekends, visiting friends, reading books, sitting in churches, using the Internet, and listening to music. The daily sitting time or ST categories were similar to those used in recent studies: ≤ 3.9, 4.0–5.9, 6.0–7.9, and ≥ 8.0 h/day [27, 28].

#### Control variables

Other covariates such as socioeconomic and health factors were also included as potential confounding variables. Socioeconomic factors included age, marital status, education level, and household income. Occupation was categorized according to the Korean version of the Standard Classification of Occupations, based on the International Standard Classification of Occupations developed by the International Labor Organization. We re-classified occupation into four categories: white collar (office work), pink collar (sales and service), blue collar (agriculture, forestry, fishery, and armed forces), and inoccupation (those with no jobs, housewives, and students). Health factors included smoking status, drinking status, walking frequency, number of chronic diseases, perceived health status, and perceived stress.

### Statistical analyses

Descriptive statistics are presented as frequencies (N) and percentages (%), and the chi-square test was used to assess significant difference in all covariates by ST and SQ categories. After adjusting for demographic and health factors, logistic regression analysis was performed to calculate the odds ratios (ORs) for all ST categories (4.0–5.9, 6.0–7.9, and ≥ 8.0 h/day) and compare the data with those of the shortest ST category (≤ 3.9 h/day). The results were reported using ORs and 95% confidence intervals (CIs). Furthermore, an ordinal logistic regression was performed to determine the association between ST categories and each component of the PSQI to assess the dose-response relationship.

All statistical analyses were performed using SAS software, version 9.4 (SAS Institute Inc., Cary, NC, USA). Statistical results were considered significant at a *p*-value of <.05.

## Results

We analyzed each of the variables stratified by sex. Table 1 shows the general characteristics of the study population. Among the 224,118 study participants, poor SQ was observed in 35,784/100,454 (35.6%) men and 58,271/123,664 (47.1%) women. As shown in Table 1, most men (32.8%) and women (39.7%) reported an ST of ≤ 3.9 h/day; the lowest frequency was observed for an ST of 6.0–7.9 h/day (17.3 and 13.1%).

**Table 1:** General characteristics of the study population

Variables	Poor sleep quality (PSQI > 5)													
	Men							Women						
	TOTAL		Yes		No		***P-value***	TOTAL		Yes		No		***P-value***
	N	%	N	%	N	%		N	%	N	%	N	%	
**Total (*N*=224,118)**	100,454	100.0	35,784	35.6	64,670	64.4		123,664	100.0	58,271	47.1	65,393	52.9	
**Sedentary time (hours/day)**							<.0001							<.0001
≤3.9	32,998	32.8	10,496	31.8	22,502	68.2		39,886	32.3	17,313	43.4	22,573	56.6	
4.0–5.9	23,450	23.3	8,194	34.9	15,256	65.1		29,745	24.1	13,794	46.4	15,951	53.6	
6.0–7.9	13,197	13.1	4,827	36.6	8,370	63.4		17,367	14.0	8,359	48.1	9,008	51.9	
≥8.0	30,809	30.7	12,267	39.8	18,542	60.2		36,666	29.6	18,805	51.3	17,861	48.7	
**Age**							<.0001							<.0001
19-29	11,181	11.1	3,358	30.0	7,823	70.0		12,034	9.7	4,538	37.7	7,496	62.3	
30-39	13,115	13.1	4,468	34.1	8,647	65.9		14,793	12.0	6,002	40.6	8,791	59.4	
40-49	17,015	16.9	5,813	34.2	11,202	65.8	<.001	19,349	15.6	7,441	38.5	11,908	61.5	<.001
50-59	19,778	19.7	6,824	34.5	12,954	65.5		23,966	19.4	10,801	45.1	13,165	54.9	
60-69	19,030	18.9	6,841	35.9	12,189	64.1		22,757	18.4	11,487	50.5	11,270	49.5	
≥70	20,335	20.2	8,480	41.7	11,855	58.3		30,765	24.9	18,002	58.5	12,763	41.5	
**Marital status**							<.0001							<.0001
Living with spouse	72,622	72.3	25,124	34.6	47,498	65.4		77,987	63.1	35,189	45.1	42,798	54.9	
Living without spouse	27,832	27.7	10,660	38.3	17,172	61.7		45,677	36.9	23,082	50.5	22,595	49.5	
**Occupational categories**^a^							<.0001							<.0001
White	22,346	22.2	7,196	32.2	15,150	67.8		20,802	16.8	7,776	37.4	13,026	62.6	
Pink	9,885	9.8	3,238	32.8	6,647	67.2		17,821	14.4	7,628	42.8	10,193	57.2	
Blue	42,023	41.8	14,687	34.9	27,336	65.1		25,394	20.5	12,097	47.6	13,297	52.4	
Inoccupation	26,200	26.1	10,663	40.7	15,537	59.3		59,647	48.2	30,770	51.6	28,877	48.4	
**Educational level**							<.0001							<.0001
Middle shool or lower	26,914	26.8	11,072	41.1	15,842	58.9		53,156	43.0	29,440	55.4	23,716	44.6	
High school	31,545	31.4	11,125	35.3	20,420	64.7		32,069	25.9	14,085	43.9	17,984	56.1	
College or higher	41,995	41.8	13,587	32.4	28,408	67.6		38,439	31.1	14,746	38.4	23,693	61.6	
**Household income**							<.0001							<.0001
Low	15,795	15.7	6,919	43.8	8,876	56.2		27,680	22.4	15,903	57.5	11,777	42.5	
Mid-low	30,637	30.5	11,529	37.6	19,108	62.4		36,380	29.4	17,961	49.4	18,419	50.6	
Mid-high	26,618	26.5	8,724	32.8	17,894	67.2		28,975	23.4	12,243	42.3	16,732	57.7	
High	27,404	27.3	8,612	31.4	18,792	68.6		30,629	24.8	12,164	39.7	18,465	60.3	
**Smoking status**							<.0001							<.0001
Current smokers	35,155	35.0	13,053	37.1	22,102	62.9		3,663	3.0	2,242	61.2	1,421	38.8	
Past smokers	37,909	37.7	14,146	37.3	23,763	62.7		2,846	2.3	1,654	58.1	1,192	41.9	
Nonsmoker	27,390	27.3	8,585	31.3	18,805	68.7		117,155	94.7	54,375	46.4	62,780	53.6	
**Drinking status**							<.0001							<.0001
Yes	67,608	67.3	23,653	35.0	43,955	65.0		46,317	37.5	20,494	44.2	25,823	55.8	
No	32,846	32.7	12,131	36.9	20,715	63.1		77,347	62.5	37,777	48.8	39,570	51.2	
**Walking frequently**^b^							<.0001							<.0001
Yes	45,799	45.6	15,599	34.1	30,200	65.9		53,681	43.4	24,092	44.9	29,589	55.1	
No	54,655	54.4	20,185	36.9	34,470	63.1		69,983	56.6	34,179	48.8	35,804	51.2	
**Obesity status (BMI)**^c^							<.0001							<.0001
Underweight & normal range	2,337	2.3	982	42.0	1,355	58.0		4,850	3.9	2,281	47.0	2,569	53.0	
Overweight	44,744	44.5	16,131	36.1	28,613	63.9		62,287	50.4	28,882	46.4	33,405	53.6	
Obese	53,373	53.1	18,671	35.0	34,702	65.0		56,527	45.7	27,108	48.0	29,419	52.0	
**The number of chronic diseases**^d^							<.0001							<.0001
0	68,031	67.7	22,909	33.7	45,122	66.3		83,451	67.5	35,941	43.1	47,510	56.9	
1	25,143	25.0	9,812	39.0	15,331	61.0		31,566	25.5	17,249	54.6	14,317	45.4	
2	7,280	7.2	3,063	42.1	4,217	57.9		8,647	7.0	5,081	58.8	3,566	41.2	
**Perceived health status**^e^							<.0001							<.0001
Yes	77,817	77.5	24,276	31.2	53,541	68.8		93,964	76.0	39,192	41.7	54,772	58.3	
No	22,637	22.5	11,508	50.8	11,129	49.2		29,700	24.0	19,079	64.2	10,621	35.8	
**Perceived stress**							<.0001							<.0001
Less	40,442	40.3	10,585	26.2	29,857	73.8		38,489	31.1	12,459	32.4	26,030	67.6	
More	60,012	59.7	25,199	42.0	34,813	58.0		85,175	68.9	45,812	53.8	39,363	46.2	

^a^ Three groups (white, pink, blue) based on the International Standard Classification Occupations codes. Inoccupation group includes housewives

^b^ Walking frequency: Based on the recommended walking volume according to the physical activity guidelines in Korea

^c^ *BMI* Body mass index/obesity status defined by BMI based on the 2018 Clinical Practice Guidelines for Overweight and Obesity in Korea

^d^ Chronic disease was defined as diagnosed diseases: hypertension, diabetes. The number of chronic diseases is the sum of the number of diagnosed above diseases the sum of the number of diagnosed above diseases

^e^ Perceived health status was classified the respondents of ‘very good’ and ‘good’ as ‘Yes’ and of ‘normal’, ‘bad’, and ‘very bad’ as ‘No’ group

Table 2 presents the factors associated with poor SQ by sex. In men, the odds of poor SQ increased with increasing ST: 4.0–5.9 h/day (OR: 1.12, 95% CI: 1.08–1.16), 6.0–7.9 h/day (OR: 1.19, 95% CI: 1.14–1.25), and ≥ 8.0 h/day (OR: 1.30, 95% CI: 1.25–1.34). Similarly, in women, the odds of poor SQ increased with increasing ST: 4.0–5.9 h/day (OR: 1.06, 95% CI: 1.03–1.10), 6.0–7.9 h/day (OR: 1.12, 95% CI: 1.08–1.16), and ≥ 8.0 h/day (OR: 1.22, 95% CI: 1.18–1.26).

**Table 2:** Association between sedentary time and poor sleep quality

Variables	Poor sleep quality (PSQI > 5)							
	Men				Women			
	OR	95% CI			OR	95% CI		
**Total**								
**Sedentary time (hours/day)**								
≤3.9	1.00				1.00			
4.0-5.9	1.12	(1.08	–	1.16)	1.06	(1.03	–	1.10)
6.0-7.9	1.19	(1.14	–	1.25)	1.12	(1.08	–	1.16)
≥8.0	1.30	(1.25	–	1.34)	1.22	(1.18	–	1.26)
**Age**								
19-29	1.00				1.00			
30-39	1.28	(1.20	–	1.36)	1.15	(1.09	–	1.22)
40-49	1.29	(1.21	–	1.37)	1.07	(1.02	–	1.13)
50-59	1.33	(1.25	–	1.42)	1.32	(1.25	–	1.40)
60-69	1.35	(1.26	–	1.44)	1.39	(1.30	–	1.47)
≥70	1.47	(1.37	–	1.58)	1.53	(1.43	–	1.63)
**Marital status**								
Living with spouse	1.00				1.00			
Living without spouse	1.36	(1.31	–	1.41)	1.08	(1.05	–	1.11)
**Occupational categories**^a^								
White	1.00				1.00			
Pink	1.02	(0.96	–	1.07)	1.07	(1.02	–	1.12)
Blue	1.07	(1.03	–	1.12)	1.08	(1.03	–	1.13)
Inoccupation	1.18	(1.12	–	1.24)	1.28	(1.23	–	1.33)
**Educational level**								
Middle shool or lower	1.00				1.00			
High school	0.91	(0.87	–	0.94)	0.89	(0.86	–	0.93)
College or higher	0.87	(0.83	–	0.92)	0.80	(0.77	–	0.84)
**Household income**								
Low	1.00				1.00			
Mid-low	0.90	(0.87	–	0.94)	0.91	(0.88	–	0.94)
Mid-high	0.82	(0.79	–	0.86)	0.85	(0.82	–	0.88)
High	0.81	(0.77	–	0.85)	0.85	(0.82	–	0.89)
**Smoking status**					1.00			
Current smokers	1.00				1.00			
Past smokers	1.00	(0.97	–	1.04)	0.90	(0.81	–	1.00)
Nonsmokers	0.87	(0.84	–	0.90)	0.64	(0.59	–	0.68)
**Drinking status**								
Yes	1.00				1.00			
No	0.95	(0.92	–	0.98)	0.90	(0.87	–	0.92)
**Walking frequently**^b^								
Yes	1.00				1.00			
No	1.03	(1.00	–	1.06)	1.05	(1.02	–	1.07)
**Obesity status (BMI)**^c^								
Underweight & normal range	1.00				1.00			
Overweight	0.97	(0.89	–	1.06)	0.99	(0.93	–	1.06)
Obese	0.92	(0.84	–	1.00)	0.96	(0.90	–	1.02)
**The number of chronic diseases**^d^								
0	1.00				1.00			
1	1.04	(1.01	–	1.08)	1.05	(1.01	–	1.08)
2	1.07	(1.02	–	1.13)	1.08	(1.03	–	1.14)
**Perceived health status**^e^								
Yes	1.00				1.00			
No	1.73	(1.68	–	1.78)	1.85	(1.80	–	1.90)
**Perceived stress**								
Less	1.00							
More	0.44	(0.43	–	0.46)	0.39	(0.38	–	0.41)

^a^ Three groups (white, pink, blue) based on the International standard classification occupations codes. Inoccupation group includes housewives

^b^ Walking frequency: Based on the recommended walking volume according to the physical activity guidelines in Korea

^c^ BMI : Body mass index/obesity status defined by BMI based on the 2018 clinical practice guidelines for overweight and obesity in korea

^d^ Chronic disease was defined as diagnosed diseases: hypertension, diabetes. The number of chronic diseases is the sum of the number of diagnosed above diseases the sum of the number of diagnosed above diseases

^e^ Perceived health status was classified the respondents of ‘very good’ and ‘good’ as ‘Yes’ and of ‘normal’, ‘bad’, and ‘very bad’ as ‘No’ group

**Fig. 1:**
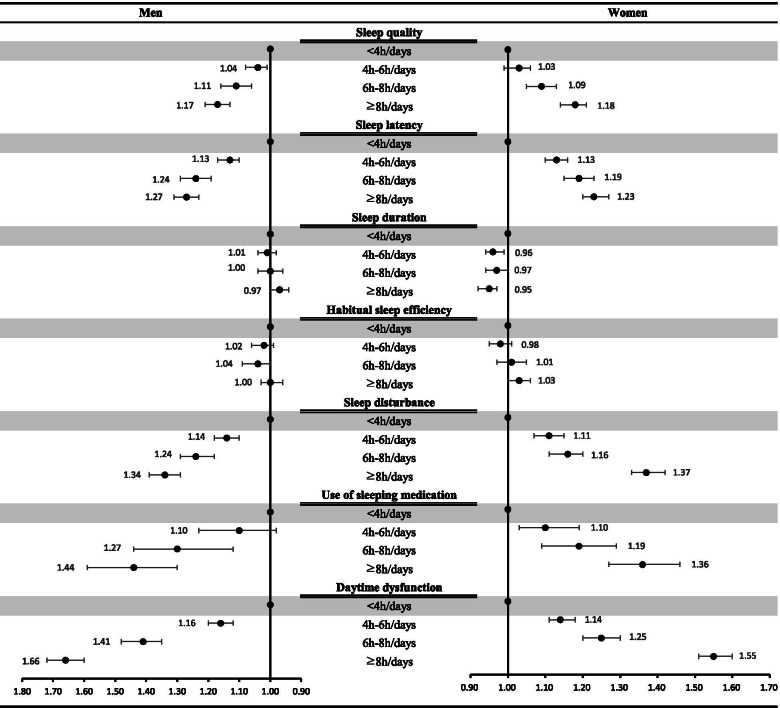
The result of subgroup analysis stratified by dependent variables using ordinal logistic regression. Adjusted by variables including Gender, Age, Marital status, Occupational, Household income, Smoking status, Drinking status, Walking frequently, obesity status (BMI), The number of chronic diseases, Perceived Health status and Perceived stress

Figure 1 shows the subgroup analyses of the association between ST and poor SQ stratified by dependent variables. As shown in Fig. 1, greater sedentary time as associated with higher odds of poor SQ across each of the components of the PSQI such as sleep quality, sleep latency, sleep disturbance, use of sleeping medication, and daytime dysfunction.

## Discussion

Our results show that more women have poor SQ than men, which is similar to those of previous studies [29, 30]. The reasons for the poorer SQ in women than in men have been explained in previous studies. Some reasons include hormonal differences due to menstruation, pregnancy and menopause [23], differences in the amounts of leisure time, socio-cultural factors due to housework [31], and the high prevalence of mental disorders in women [32].

Our study results suggest there is a positive association between sedentary time and sleep problems. Several studies also demonstrate this association [30]. Furthermore, several mechanisms according to these associations have also been proposed [16, 33–36]. A potential mechanism for this is that sleep time can be impaired by the amount of time spent exercising certain behaviors, given that ST is usually associated with watching television or computer use [16, 33]. Insufficient sleep time can be a factor that naturally lowers the quality of sleep [30]. Increased ST, such as from watching television and computer use, may increase the risk of mental health problems by promoting social isolation and limiting development of social network [34, 37]. Consequently, increased ST is associated with mental health problems such as depression, which may contribute to poor SQ [38–40]. Furthermore, light-emitting diode (LED)-backlit displays are increasingly used in TV and computer screens. The LED-backlit display may cause significant suppression of melatonin, thus affecting the biological clock and possibly resulting in sleep problems [35]. In addition, sedentary behavior also contributes to the onset and progression of metabolic syndrome, which can cause sleep problems [41]. Moreover, compared to sedentary behavior, nonsedentary behavior is associated with increased energy expenditure/metabolic rate and fatigue, which may reduce the risk of sleep problems [42, 43]. Regardless of achieving sufficient physical activity, standing behavior alone can cope with this [2, 41].

In our study, from the subgroup analysis stratified by the seven items of PSQI, sleep duration and habitual sleep efficiency were not dose-response related to ST. This result is consistent with those of previous studies that defined ST as a comprehensive state and that examined the relationship between sleep duration and ST [16]. However, the result is inconsistent previous studies that examined the association of ST with sleep duration by focusing on screen time [44]. Therefore, because our study included all relevant activities while defining ST, including reading books, writing, and watching television, it seems that there was no difference in the relationship between ST and sleep duration. Additionally, previous studies showed different results for habitual sleep efficiency depending on how ST was defined [30]. Therefore, more in-depth research is needed to examine the relationship of ST with sleep duration and habitual sleep efficiency.

Although the results of this study can play an important role in clarifying how ST is associated with poor SQ, it has some limitations. First, we used cross-sectional data; thus, we could only determine the association between ST and SQ. We could not investigate the causal relationship between these two variables. According to a recent study, poor sleep quality for one night impacts daily life the following day, including increased sedentary time. As such, there is a possibility of a similar observation seen in the reverse direction [45]. Further explanatory research is needed to infer causality. Second, the sub-category measures of ST and SQ were self-reported. As such, these measures were dependent on participants’ ability to recall, and the responses might not have been accurate. Third, ST was broadly defined and investigated. ST is not specific because it was deduced from the response to one question. Therefore, to examine the relationship between ST and SQ more precisely, detailed investigation of ST is needed in further studies.

Despite these limitations, our research has several advantages. As this study was conducted using a large sample, the results can be considered representative of the Korean population. Our study is meaningful because unlike previous studies, ST was comprehensively investigated and analyzed without focusing only on-screen time (watching TV and using the Internet). Furthermore, unlike previous studies, we used the PSQI, which is a useful tool that can comprehensively examine SQ.

## Conclusions

This study confirmed that both men and women were at risk of the threat of poor SQ due to prolonged ST. Replacing ST with light-intensity physical activity or even standing time may have a positive effect on health [2, 18]. Therefore, to reduce ST and to promote healthy behaviors, the following points should be considered: As mental health-related studies involving ST and SQ are scarce, qualitative and quantitative research is needed to identify sedentary behavior patterns and to investigate their causal relationships with health status according to the types and classifications of sedentary behaviors. Furthermore, based on the results of these studies, national programs will be needed, such as those for developing ST guidelines, which can then be used to develop and evaluate health programs. Additionally, the government should identify measures to limit excessive exposure to LED screens so that healthy leisure activities can be adopted to reduce ST.

## Data Availability

Publicly available datasets were analyzed in this study. These data can be found here: [https://chs.kdca.go.kr/chs/main.do] (accessed on 6 May 2021).

## Abbreviations

CI: Confidence interval
KCHS: Korea Community Health Survey
LED: Light-emitting diode
OR: Odds ratio
PQSI: Pittsburgh Sleep Quality Index
SQ: Sleep quality
ST: Sedentary time

## References

[1] Stranges S, Tigbe W, Gómez-Olivé FX, Thorogood M, Kandala N-B (2012). Sleep problems: an emerging global epidemic? Findings from the INDEPTH WHO-SAGE study among more than 40,000 older adults from 8 countries across Africa and Asia. Sleep..

[2] Stamatakis E, Rogers K, Ding D, Berrigan D, Chau J, Hamer M (2015). All-cause mortality effects of replacing sedentary time with physical activity and sleeping using an isotemporal substitution model: a prospective study of 201,129 mid-aged and older adults. Int J Behav Nutr Phys Act.

[3] Buysse DJ, Reynolds CF, Monk TH, Berman SR, Kupfer DJ (1989). The Pittsburgh Sleep Quality Index: a new instrument for psychiatric practice and research. Psychiatry Res.

[4] Buysse DJ (2014). Sleep health: can we define it? Does it matter?. Sleep..

[5] Seow LSE, Tan XW, Chong SA, Vaingankar JA, Abdin E, Shafie S (2020). Independent and combined associations of sleep duration and sleep quality with common physical and mental disorders: results from a multi-ethnic population-based study. PLoS One.

[6] Altevogt BM, Colten HR (2006). Sleep disorders and sleep deprivation: an unmet public health problem.

[7] Ahn DH (2013). Insomnia: causes and diagnosis. Hanyang Med Rev.

[8] Kripke DF, Klauber MR, Wingard DL, Fell RL, Assmus JD, Garfinkel L (1998). Mortality hazard associated with prescription hypnotics. Biol Psychiatry.

[9] Youngstedt SD (2003). Ceiling and floor effects in sleep research. Sleep Med Rev.

[10] Youngstedt SD (2005). Effects of exercise on sleep. Clin Sports Med.

[11] Kim MJ, Shin JH, Kim CS, Lee MS, Jeong UH (2015). Related-factors of sleep quality among some adults. Korean J Fam Pract.

[12] Tremblay MS, Colley RC, Saunders TJ, Healy GN, Owen N (2010). Physiological and health implications of a sedentary lifestyle. Appl Physiol Nutr Metab.

[13] Van der Ploeg HP, Chey T, Korda RJ, Banks E, Bauman A (2012). Sitting time and all-cause mortality risk in 222 497 Australian adults. Arch Intern Med.

[14] Sherrill DL, Kotchou K, Quan SF (1998). Association of physical activity and human sleep disorders. Arch Intern Med.

[15] Aadahl M, Andreasen AH, Hammer-Helmich L, Buhelt L, Jorgensen T, Glümer C (2013). Recent temporal trends in sleep duration, domain-specific sedentary behaviour and physical activity. A survey among 25–79-year-old Danish adults. Scand J Public Health.

[16] Lakerveld J, Mackenbach J, Horvath E, Rutters F, Compernolle S, Bárdos H (2016). The relation between sleep duration and sedentary behaviours in European adults. Obes Rev.

[17] Lallukka T, Sivertsen B, Kronholm E, Bin YS, Overland S, Glozier N (2018). Association of sleep duration and sleep quality with the physical, social, and emotional functioning among Australian adults. Sleep Health.

[18] Seol J, Abe T, Fujii Y, Joho K, Okura T (2020). Effects of sedentary behavior and physical activity on sleep quality in older people: a cross-sectional study. Nurs Health Sci.

[19] Mollayeva T, Thurairajah P, Burton K, Mollayeva S, Shapiro CM, Colantonio A (2016). The Pittsburgh sleep quality index as a screening tool for sleep dysfunction in clinical and non-clinical samples: a systematic review and meta-analysis. Sleep Med Rev.

[20] Sohn SI, Kim DH, Lee MY, Cho YW (2012). The reliability and validity of the Korean version of the Pittsburgh Sleep Quality Index. Sleep Breath.

[21] Wu X, Tao S, Zhang Y, Zhang S, Tao F (2015). Low physical activity and high screen time can increase the risks of mental health problems and poor sleep quality among Chinese college students. PLoS One.

[22] Kang YW, Ko YS, Kim YJ, Sung KM, Kim HJ, Choi HY (2015). Korea community health survey data profiles. Osong Public Health Res Perspect.

[23] Andersen ML, Alvarenga TF, Mazaro-Costa R, Hachul HC, Tufik S (2011). The association of testosterone, sleep, and sexual function in men and women. Brain Res.

[24] Choi H, Kim S, Kim B, Kim I (2015). Psychometric properties of the Korean versions of three sleep evaluation questionnaires. Clin Nurs Res.

[25] Lee SY, Ju YJ, Lee JE, Kim YT, Hong SC, Choi YJ (2020). Factors associated with poor sleep quality in the Korean general population: providing information from the Korean version of the Pittsburgh Sleep Quality Index. J Affect Disord.

[26] Park SK, Jung JY, Oh C-M, McIntyre RS, Lee J-H (2018). Association between sleep duration, quality and body mass index in the Korean population. J Clin Sleep Med.

[27] Stamatakis E, Gale J, Bauman A, Ekelund U, Hamer M, Ding D (2019). Sitting time, physical activity, and risk of mortality in adults. J Am Coll Cardiol.

[28] Ekelund U, Steene-Johannessen J, Brown WJ, Fagerland MW, Owen N, Powell KE (2016). Does physical activity attenuate, or even eliminate, the detrimental association of sitting time with mortality? A harmonised meta-analysis of data from more than 1 million men and women. Lancet.

[29] Tang J, Liao Y, Kelly BC, Xie L, Xiang Y-T, Qi C (2017). Gender and regional differences in sleep quality and insomnia: a general population-based study in Hunan Province of China. Sci Rep.

[30] Yang Y, Shin JC, Li D, An R (2017). Sedentary behavior and sleep problems: a systematic review and meta-analysis. Int J Behav Med.

[31] Rich AJ, Koehoorn M, Ayas NT, Shoveller J. Gender/sex disparity in self-reported sleep quality among Canadian adults. UBC Med J. 2020;11(2):11–13.

[32] Chandra PS, Satyanarayana VA (2010). Gender disadvantage and common mental disorders in women. Int Rev Psychiatry.

[33] Van den Bulck J (2004). Television viewing, computer game playing, and Internet use and self-reported time to bed and time out of bed in secondary-school children. Sleep..

[34] Ancoli-Israel S (2009). Sleep and its disorders in aging populations. Sleep Med.

[35] Bues M, Pross A, Stefani O, Frey S, Anders D, Späti J (2012). LED-backlit computer screens influence our biological clock and keep us more awake. J Soc Inf Disp.

[36] Wolk R, Somers VK (2007). Sleep and the metabolic syndrome. Exp Physiol.

[37] Buman MP, Kline CE, Youngstedt SD, Phillips B, De Mello MT, Hirshkowitz M (2015). Sitting and television viewing: novel risk factors for sleep disturbance and apnea risk? Results from the 2013 National Sleep Foundation Sleep in America Poll. Chest..

[38] Teychenne M, Abbott G, Ball K, Salmon J (2014). Prospective associations between sedentary behaviour and risk of depression in socio-economically disadvantaged women. Prev Med.

[39] Brunet J, Sabiston CM, O’Loughlin E, Chaiton M, Low NC, O’Loughlin JL (2014). Symptoms of depression are longitudinally associated with sedentary behaviors among young men but not among young women. Prev Med.

[40] Gregory AM, Rijsdijk FV, Lau JY, Dahl RE, Eley TC (2009). The direction of longitudinal associations between sleep problems and depression symptoms: a study of twins aged 8 and 10 years. Sleep..

[41] Sisson SB, Camhi SM, Church TS, Martin CK, Tudor-Locke C, Bouchard C (2009). Leisure time sedentary behavior, occupational/domestic physical activity, and metabolic syndrome in US men and women. Metab Syndr Relat Disord.

[42] Morselli LL, Guyon A, Spiegel K (2012). Sleep and metabolic function. Pflügers Archiv.

[43] Uchida S, Shioda K, Morita Y, Kubota C, Ganeko M, Takeda N (2012). Exercise effects on sleep physiology. Front Neurol.

[44] Lovro Š, Horvatin M, Baić M (2019). Are sedentary behaviors associated with sleep duration? A cross-sectional case from Croatia. Int J Environ Res Public Health.

[45] Thosar SS, Bhide MC, Katlaps I, Bowles NP, Shea SA, McHill AW (2021). Shorter sleep predicts longer subsequent day sedentary duration in healthy midlife adults, but not in those with sleep apnea. Nat Sci Sleep.

